# Supplementary material for: Patterns of Intron Gain and Loss in Fungi
Source: PLoS Biol. 2004 Nov 30;2(12):e422. doi: 10.1371/journal.pbio.0020422 (PMC532390; doi:10.1371/journal.pbio.0020422)
Supplement: Table S1 — Also available at http://genes.mit.edu/NielsenEtAl/. (4.3 MB ZIP). [file pbio.0020422.st001.zip › NielsenEtAl/html/1199.html]

AN4804.1.NCU01971.1.MG02950.1.FG08501.1


```
 CLUSTAL W (1.82) Multiple Sequence Alignments - Introns Inserted


Sequence 1: MG02950.1	291 aa
Sequence 2: FG08501.1	347 aa
Sequence 3: NCU01971.1	343 aa
Sequence 4: AN4804.1	298 aa
Alignment Length: 425 aa
Number Identitical Residues: 88 aa
Alignment Score (without introns) 4657


MG02950.1 	----------MDEK----------------------TAKVVAQIDSLSSKR-----ELTI
NCU01971.1	MTPRTTTPRTMTPRARTTRATTPRPGSSDAAALLQRAAEVLQALGSLDASTRD---EITI
FG08501.1 	----------MGQPD---------------------AQKVLDTLTKLQSTQ-----EISI
AN4804.1  	----------MAEPN---------------------AAQIRQQIKQLEQEHGELKTQLAI
          	          *                         : ::   : .*.    . .::::*

MG02950.1 	ISLAEPITSAVEQSEANGSGS-------------NNNVRTSDIS----TASLDAPTPASL
NCU01971.1	IKISEPISSALHSAASKNSSSNSGNDESHNQQPSTAAPRTSDVSAHSASSALDAPTPASL
FG08501.1 	IKLSEPIANSNPQ---------------------DARQRTSDAS----NSAFDALTPSSL
AN4804.1  	VRISEPIFSPDTDR--------------------SPSKRNSDVS------TVDSPSPASL
          	: ::*** ..  .                         *.** *      :.*: :*:**

MG02950.1 	EADLEHYK~ELFAKLRFSYVEQVTKEKFIRAIVGDPPLIVDPSENAELEAGNAAVKAELK
NCU01971.1	EADLAHYK~ELFAKLRFSYVEQVTKEKFIRAIVGDPPLIVTPQENADLEASNMAAKASLK
FG08501.1 	TADLAHYK~ELFAKLRFSYVQQVTKEKFIRAIVGDPPMIVSMEENLELEKENMVVKKQLK
AN4804.1  	EADLSHYK0ELFSKLRFSYVEQVTKEKFLRAIVGDPPLVVGHNENVELEAQLAEVKAELK
          	 *** *** ***:*******:*******:********::*  .** :**     .* .**

MG02950.1 	ALKREVEALIAELERKGADLARR~--------------QEKIELERVQLAEMPGRIGGLE
NCU01971.1	ALKTEVALLAEDLESRSRELAAR~--------------YERVKLDRIKLRELPGKMEELQ
FG08501.1 	ELKTEVAEMVTDLERRGIELSKK~--------------YESVQFETAELREMPAKIEELE
AN4804.1  	ARKEEVRLMVEEMEKMARDLATR1SRGKGVSNDSRLSGYNNVQLQMTQLATLPESIENLE
          	  * **  :  ::*  . :*: : : ... :..:  :. : ::::  :*  :*  :  *:

MG02950.1 	EAVAELRSRSNELAEGGSSDMRLPLAKTVALVEQRAAEVRELDRRLETLKAAAPRKRKEM
NCU01971.1	KRVDKLRT-KQAIQPGSQPEMNLPLAKTAVLVEERRQKVRELERQVEQLSSLAPRKRKEM
FG08501.1 	ARIEILRE-EQEMPDGTSPGMNLPLAKTQDLVNQRKLEQQELARELESLQAKVPRKRKEA
AN4804.1  	STIAALRA-KQIANSETSSSQNLPLPATLSLLAKREAELAALNRQIAAAQNTLPRKTREA
          	  :  **  .:      ..  .***. *  *: :*  :   * *.:   .   *** :* 

MG02950.1 	ERLQAELVPLEAKRQNSTAAAKEAKRRKE--AALGGAEDDLEERGRWWRTSE-~------
NCU01971.1	ERLQMEVTALAAKRAQVSAAAREAKRRRENAQKAGGTYDEMEAKGRWYRASE-~------
FG08501.1 	DRLLIELQPLENKRQNSATAAREARRRKE--AALGGAADDLEERARWWRASEG1AELYLD
AN4804.1  	ETMERELGILERRKSEAIMQAREAQRKKQ-----EGESDGLEEAGRWYRSAE-~------
          	: :  *:  *  :: :    *:**:*:::      *  * :*  .**:*::*        

MG02950.1 	----ALLKSMLE~----------IKN----------------------------------
NCU01971.1	----AALREMLG~----------LKE----------------------------------
FG08501.1 	EASMALASTVIH1NPAAVLKEESVKNKMIYLAIPVCKRMDTDALVAILTGNEHWIDFSFV
AN4804.1  	----EALKKLLG~----------VQG----------------------------------
          	         ::            ::                                   

MG02950.1 	---------
NCU01971.1	---------
FG08501.1 	SCCLDVGVE
AN4804.1  	---------
          	
```
